# Wheat ATIs: Characteristics and Role in Human Disease

**DOI:** 10.3389/fnut.2021.667370

**Published:** 2021-05-28

**Authors:** Sabrina Geisslitz, Peter Shewry, Fred Brouns, Antoine H. P. America, Giacomo Pietro Ismaele Caio, Matthew Daly, Stefano D'Amico, Roberto De Giorgio, Luud Gilissen, Heinrich Grausgruber, Xin Huang, Daisy Jonkers, Daniel Keszthelyi, Colette Larré, Stefania Masci, Clare Mills, Marie Sofie Møller, Mark E. Sorrells, Birte Svensson, Victor F. Zevallos, Peter Louis Weegels

**Affiliations:** ^1^Department of Bioactive and Functional Food Chemistry, Institute of Applied Biosciences, Karlsruhe Institute of Technology (KIT), Karlsruhe, Germany; ^2^Rothamsted Research, Harpenden, United Kingdom; ^3^Department of Human Biology, Faculty of Health, Medicine and Life Sciences, School for Nutrition and Translational Research in Metabolism (NUTRIM), Maastricht University, Maastricht, Netherlands; ^4^BU Bioscience, Plant Sciences Group, Wageningen University and Research, Wageningen, Netherlands; ^5^Department of Morphology, Surgery and Experimental Medicine, St. Anna Hospital, University of Ferrara, Ferrara, Italy; ^6^Division of Infection, Immunity and Respiratory Medicine, Manchester Institute of Biotechnology, University of Manchester, Manchester, United Kingdom; ^7^Institute for Animal Nutrition and Feed, AGES - Austrian Agency for Health and Food Safety, Vienna, Austria; ^8^Wageningen University and Research, Plant Breeding, Wageningen, Netherlands; ^9^Department of Crop Sciences, University of Natural Resources and Life Sciences, Vienna, Austria; ^10^Department of Food and Nutrition, Faculty of Agriculture and Forestry, University of Helsinki, Helsinki, Finland; ^11^Division of Gastroenterology-Hepatology, Department of Internal Medicine and School for Nutrition and Translational Research in Metabolism (NUTRIM), Maastricht University Medical Centre, Maastricht, Netherlands; ^12^INRAE UR1268 BIA, Impasse Thérèse Bertrand-Fontaine, Nantes, France; ^13^Department of Agriculture and Forest Sciences, University of Tuscia, Via San Camillo de Lellis, Viterbo, Italy; ^14^Enzyme and Protein Chemistry, Department of Biotechnology and Biomedicine, Technical University of Denmark, Lyngby, Denmark; ^15^School of Integrative Plant Science, Plant Breeding and Genetics Section, Cornell University, Ithaca, NY, United States; ^16^Nutrition and Food Research Group, Department of Applied and Health Sciences, University of Northumbria, Newcastle Upon Tyne, United Kingdom; ^17^Laboratory of Food Chemistry, Wageningen University and Research, Wageningen, Netherlands

**Keywords:** wheat, amylase/trypsin-inhibitors, health, pathology, food technology, genetics

## Abstract

Amylase/trypsin-inhibitors (ATIs) comprise about 2–4% of the total wheat grain proteins and may contribute to natural defense against pests and pathogens. However, they are currently among the most widely studied wheat components because of their proposed role in adverse reactions to wheat consumption in humans. ATIs have long been known to contribute to IgE-mediated allergy (notably Bakers' asthma), but interest has increased since 2012 when they were shown to be able to trigger the innate immune system, with attention focused on their role in coeliac disease which affects about 1% of the population and, more recently, in non-coeliac wheat sensitivity which may affect up to 10% of the population. This has led to studies of their structure, inhibitory properties, genetics, control of expression, behavior during processing, effects on human adverse reactions to wheat and, most recently, strategies to modify their expression in the plant using gene editing. We therefore present an integrated account of this range of research, identifying inconsistencies, and gaps in our knowledge and identifying future research needs.

**Note**

This paper is the outcome of an invited international ATI expert meeting held in Amsterdam, February 3-5 2020

## Introduction

**Figure 1:**
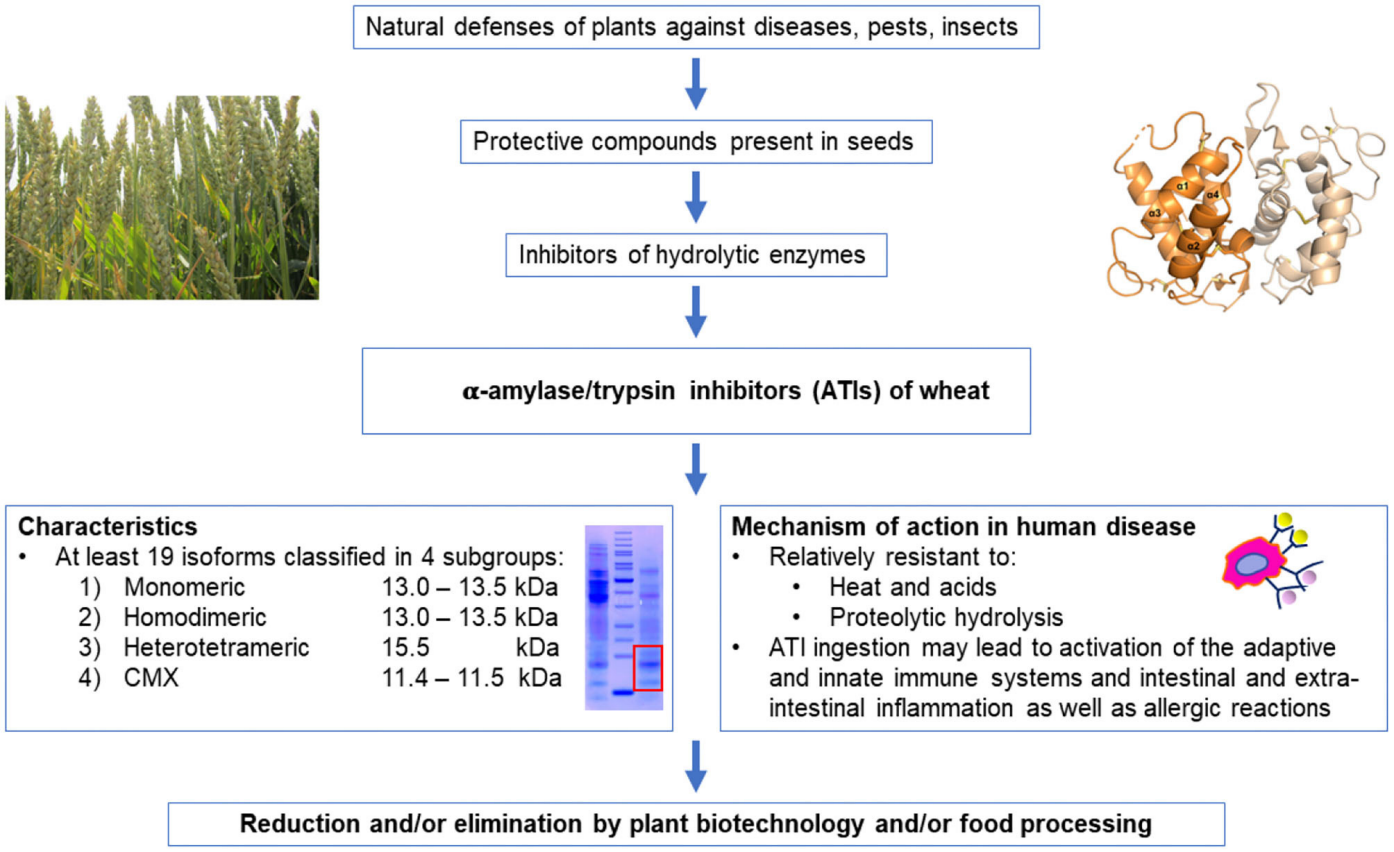
Condensed overview of the presence and key-characteristics of ATIs in wheat and their potential effects in the human body.

This paper reviews our current knowledge of ATIs from genetical, structural, functional, and health perspectives (Figure 1).

### Importance of Wheat-Based Foods

Wheat is the staple food in Europe, North Africa, West and Central Asia and most of North and South America with about 750 million tons being harvested annually (http://www.fao.org/faostat/en/#data/QC). About 95% is modern hexaploid bread wheat (*Triticum aestivum* L subsp. *aestivum*, genome constitution ABD) with most of the remaining 5% being tetraploid pasta wheat (*T. turgidum* L. subsp. *durum*, AB genomes). In addition, small amounts of traditional types of wheat, i.e., diploid einkorn (*T. monococcum* L. subsp. *monococcum*, A^m^ genome), tetraploid emmer (*T. turgidum* L. subsp. *dicoccum* Thell., AB genomes), or Khorasan wheat (*T. turanicum* Jakubz., AB genomes), and hexaploid spelt (*T. aestivum* L. subsp. *spelta* Thell., ABD genomes) are grown either for the production of traditional foods or because of perceived health benefits.

Wheat-based foods provide 20–50% of the daily intake of dietary calories in diets and contribute substantially to intakes of protein, fiber, vitamins, and minerals (1). The wheat grain typically contains about 10–15% protein of which 70–80% is gluten, a mixture of between 50 and 100 different proteins which form a visco-elastic network in dough. Gluten provides cohesion to dough and enables the entrapment of carbon dioxide produced during fermentation, resulting in expansion of the dough and the light porous crumb structure of bread. The unique properties of gluten therefore underpin the use of wheat in food processing and have contributed to the dominance of wheat-based foods in temperate countries.

The non-gluten proteins comprise a mixture of components with structural, metabolic, and putative protective functions (2). The latter include proteins which inhibit hydrolytic enzymes of pest insects and pathogenic fungi, notably the amylase/trypsin inhibitors (widely referred to as ATIs) which account for 2–4% of the total wheat protein (3). ATIs were first reported in the 1940s (4) and had been the subject of over 70 papers by the mid-1970s (5). They have well-established roles in allergic responses to wheat (as discussed below), but there has been increased interest over the past few years because they have been suggested to contribute to the development of coeliac disease (CD) in genetically susceptible individuals, affecting about a mean of 1% of the Western population. In addition, ATIs have been proposed to play a role in non-coeliac wheat sensitivity (NCWS), which has an estimated prevalence between 1 and 10% of the population, being significantly higher in women (6) than in men, and mainly based on self-diagnosis (7). The remainder of the population tolerates wheat consumption without problems. An important challenge for the study of ATIs in CD and NCWS is the lack of well-characterized protein preparations for testing. For example, it has recently become acknowledged that gluten preparations that are assumed to be pure are frequently used *in vitro* studies, animal studies and human studies addressing adverse reactions to gluten, also contain substantial amounts of other protein components, including ATIs. In addition, isolated ATI fractions used to study *in vitro* bioactivity, contain unidentified proteins which could contribute to the observed effects (8). Accordingly, as long as pure ATIs of known composition are not available, it cannot be excluded that these compounds may also play a role in the observed responses. Insight in the gaps in our knowledge and related challenges for future research are crucial in this respect.

### ATIs are Members of the Prolamin Superfamily

**Figure 2:**
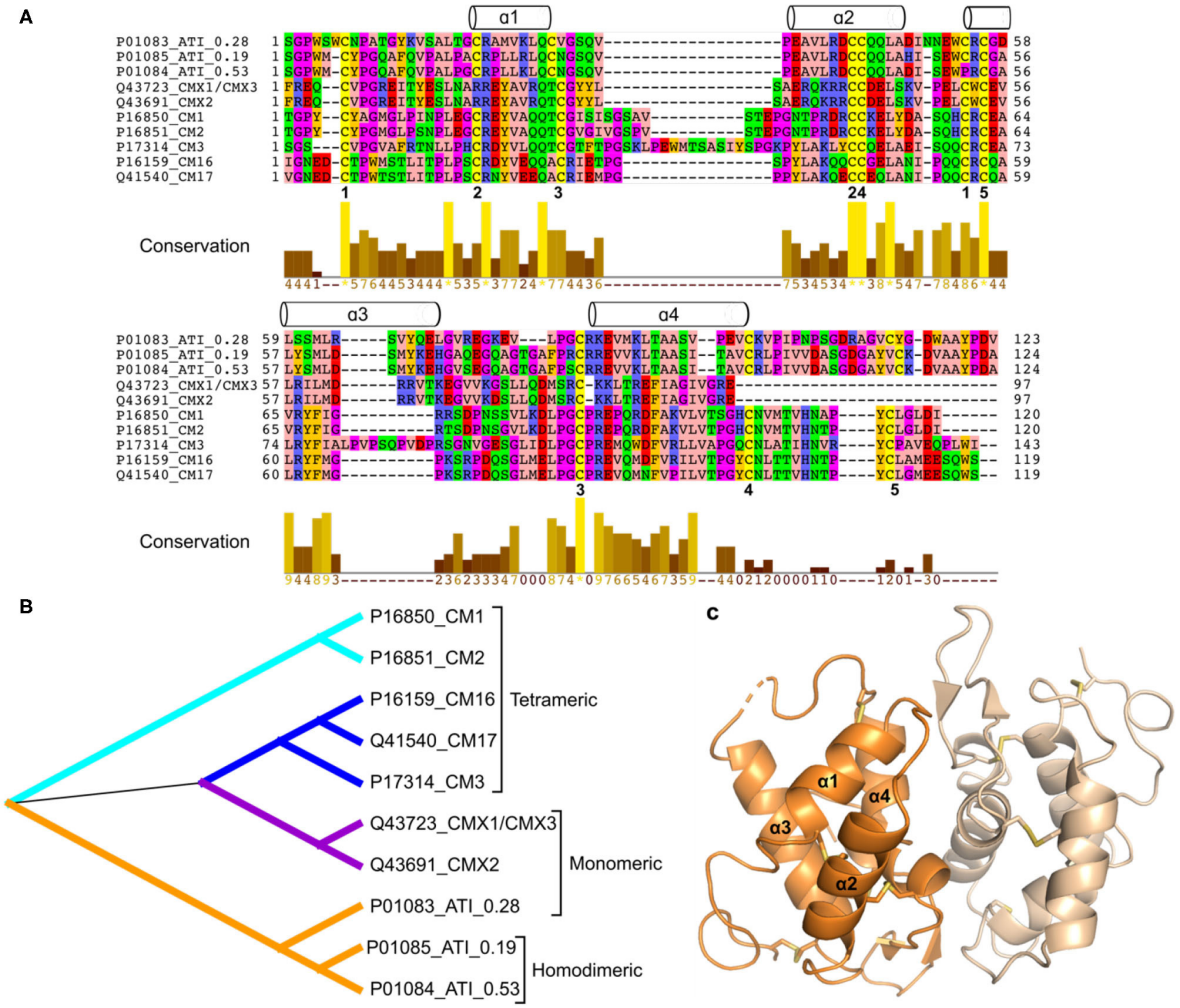
**(A)**, Multiple sequence alignment including wheat ATIs (signal peptides have been removed). The wheat ATIs only share very few fully conserved positions including some of the disulphide bonds (numbered 1–5 below the alignment). The α-helices above the alignment indicate the secondary structure elements based on the 3D structure of ATI 0.19 (part **C**), and the alignment is colored according to amino acid properties. **(B)**, Phylogenetic tree generated based on alignment shown in **(A)**. **(C)**, Three-dimensional structure of wheat homodimeric ATI 0.19 [PDB entry 1HSS (11)]. Although only one 3D structure has been determined for 0.19, and the sequence identity is low, all wheat ATIs are predicted to share the following overall structure: four α-helices connected by irregular loop regions and stabilized by disulphide bonds (a four-α-helix bundle). Software used: MEGA X (12) (Muscle for alignment preparation and Maximum likelihood for phylogenetic analysis), Dendroscope (13), Jalview (14), and PyMOL (Schrödinger, LLC).

Wheat gluten proteins, and related storage proteins from other cereal grains, are defined as prolamins because of their solubility in alcohol-water mixtures (9). Although prolamins were long thought to be unique, comparisons of amino acid sequences showed that they are related to several groups of small sulfur-rich proteins and are together defined as the “prolamin superfamily” of plant proteins (10). They include ATIs and puroindolines in cereal seeds, non-specific lipid transfer proteins in many plant tissues and *2S* storage globulins present in seeds of dicotyledonous plants. These proteins are characterized by having low molecular weights, high stability to digestion and denaturation and a conserved pattern of intrachain disulphide bonds. Although the sequence identity between the conserved regions from different members of the family is low (Figure 2A), their 3D structures are highly similar, consisting of bundles of α-helices stabilized by disulphide bonds (15). This structure is illustrated in Figure 2C.

## Types and Properties of ATIs in Bread Wheat

At least 19 isoforms of ATI have been described (16) which are classified into four groups (Table 1). The first group comprises monomeric inhibitors with the major form named 0.28 (based on its electrophoretic mobility) while the second group includes the two homodimeric inhibitors called 0.19 and 0.53. These proteins have masses between 13,000 and 13,500. The third group comprises heterotetrameric inhibitors which were originally defined as CM proteins (CM1, CM2, CM3, CM16, CM17) based on their solubility in chloroform:methanol mixtures (18). They have at the subunit level similar masses to the monomeric and dimeric types, except for CM3 which has a mass of about 15,500. However, most of our knowledge is based on the analysis of a small number of genotypes of wheat and we know little about the extent of variation in the sequences of components between genotypes.

**Table 1:** Nomenclature, amylase inhibitory activity, and abundance of wheat ATIs.

**Aggregation state**	**Protein subunit**	**Widely used names**	**Amylase inhibitory activity**	**% total flour protein**
**Monomeric**	WMAI-1	0.28	human and insects	0.5
**Homodimer**	WDAI-1	0.53	human and insects	1.0
	WDAI-2	0.19		
**Tetramer** 1st subunit	WTAI-CM1	CM1	human and insects	1.7
	WTAI-CM2	CM2		
2nd subunit	WTAI-CM16	CM16		
	WTAI-CM16 glycosylated form	CM16		
	WTAI-CM17	CM17		
3rd subunit (2 copies)	WTAI-CM3	CM3		
**Monomeric**	CMx1/2/3	Trypsin inhibitors	No known activity to amylases	0.2

*The table is based on Carbonero and Garcia-Olmedo (17) and includes data from Dupont et al. (3) and Altenbach et al. (16)*.

All of these proteins inhibit exogenous (i.e., non-wheat) α-amylases from insect and mammalian sources but their precise specificities differ. Monomeric inhibitor 0.28 has high activity against amylases from insect pests (the Coleopteran beetle *Tenebrio molitor* and larvae of the moth *Ephestia kuehniella*) but is less active against human salivary amylase (19). The dimeric 0.19 and 0.58 inhibitors are both active against human salivary and porcine pancreatic amylases, although the 0.53 inhibitor shows less activity against the porcine enzyme, and against enzymes from a range of insects, while the tetrameric inhibitors have been reported to be more active against the Lepidopteran α-amylases than the monomeric and dimeric inhibitors (19–21). They also inhibit papain, bovine trypsin, and subtilisin (22) with one monomer being able to inhibit one molecule of α-amylase and one molecule of trypsin at the same time (23).

Finally, wheat also contains inhibitors of trypsin only which have masses between 11,400 and 11,500 and are termed CMX based on their homology with the barley trypsin inhibitor. Proteomics studies have shown that at least some of the subunits occur in isoforms. For example, sequences corresponding to 15 distinct ATI proteins, comprising two monomeric, four dimeric, and six tetrameric subunits and three forms of CMX were identified in a single bread wheat variety (16). Subunit CM16 also occurs in non-glycosylated and glycosylated forms (24). More recently, Bose et al. (25) identified 33 ATI-like proteins in bread wheat using untargeted LC-MS/MS and developed a targeted LC-MS/MS method for 63 peptides that covered 18 different ATI variants. Many of the selected peptides occurred in multiple ATI variants and were also overlapping between different classes of ATI showing the complexity of ATI isoforms.

Phylogenetic analysis based on amino acid sequences shows that the monomeric and dimeric forms cluster together, with the tetrameric subunits and trypsin inhibitors forming separate clusters (Figure 2B).

Carbonaro et al. (26) proposed a unified system to name and classify ATIs based on their activities and whether they are present in the plant as monomers, dimers or tetramers (Table 1). Although this classification was an important advance in understanding the complexity of the protein family, it has not been widely adopted with most studies using a combination of the mobility-based and CM classifications.

ATIs accumulate in the grain from about seven days after pollination until maturity, but there is a small time lag before inhibitory activity is detected which may be due to the time required for subunit assembly (27). They are primarily located in the starchy endosperm and therefore enriched in white flour derived from this tissue by milling (28, 29). They are secretory proteins, being synthesized with *N*-terminal signal peptides which direct the nascent chains into the lumen of the endoplasmic reticulum. They are presumed to be located in the vacuoles of developing grain cells, but the starchy endosperm cells die during the later stages of grain development and their contents merge. Hence, ATIs may become associated with starch or gluten preparations made from flour. Although there is no evidence for specific binding of ATIs to starch, two proteins corresponding to CM16 and CM3 were identified as tightly bound to gluten proteins. They were called DSG (durum wheat sulfur-rich glutenin) and it was suggested that they contributed to dough quality (30).

## Roles of ATIs in Crop Resistance to Pests and Pathogens

Cereal grains are attractive to pests and pathogens because they have high contents of storage reserves (starch and protein). They have therefore evolved to contain a range of proteins which inhibit the hydrolytic enzymes of these organisms, including ATIs able to inhibit α-amylases from Lepidopteran and Coleopteran insects (Table 1). They are also able to inhibit a range of proteases (as discussed above), but effects on other hydrolases (such as xylanases, glucanases, and lipases) have not been determined.

ATIs are defined as pathogenesis-related (PR) proteins, being part of a group of proteins which are induced in response to damage or infection (31). They would be expected to contribute to defense against pathogens which can have significant impacts on wheat yields and quality (32). In fact, the contents of ATIs in the wheat endosperm increase by 3–10-fold during grain development, compared with an average increase of three times for other defensive proteins (33) while the amounts of the tetrameric CM1, CM3, CM17 also increase with abiotic stresses such as drought and heat (34).

*Fusarium graminearum* (Fusarium head blight), *Blumeria graminis* f.sp. *tritici* (powdery mildew) and *F. culmorum* (seedling blight, head blight, and foot rot) are important wheat diseases. Infection of wheat with these pathogens has been variously reported to result in increased contents of some ATI isoforms (35), no change in ATIs (36) or a decrease in other isoforms (37). *Fusarium*-resistant wheat had higher contents of monomeric 0.28 and one dimeric 0.19 isoform, but resistance was not related to the levels of other ATIs (37). Hence, no consistent effects of pathogen infection on ATI accumulation or amount are observed in *Fusarium*-resistant wheat. There are no strong correlations between activity or content of ATIs and presence of pathogens and no specific wheat metabolic pathways that are induced by biotic and abiotic stress and that are linked to ATIs. It is possible that the mode of action of ATIs on fungi is more complex as described for other species (19) or there are synergistic PR effects as described in barley for thionins, barley trypsin inhibitor and Bowman Birk trypsin inhibitor (38).

## Genetic and Environmental Control ATIs in Bread Wheat

The genes encoding ATIs were initially mapped to individual chromosomes and chromosome arms of hexaploid bread wheat by analyzing genetic stocks (especially using lines which differ in their complements of chromosomes, such as deletion lines). Most ATIs are encoded by the B and D genomes with genes encoding the monomeric inhibitors (0.28) on the short arms of chromosomes 6B and 6D and the dimeric 0.19 and 0.53 inhibitors on the short arms of chromosomes 3B and 3D. The subunits of the tetrameric inhibitors are encoded by genes on chromosomes 4B, 4D, 7B, and 7D. The trypsin inhibitor CMX is reported to be encoded by genes on the group 4 chromosomes of all three genomes.

Gene locations have since been determined by multiple sequence alignment using the wheat reference genome (25, 39). This essentially confirmed the earlier mapping with monomeric and dimeric ATIs being mapped to chromosomes 6 and 3, respectively, and tetrameric types to chromosomes 4 (CM3, CM16, CM17) and 7 (CM1, CM2). Additionally, some ATI genes were annotated to group 2 chromosomes (39). It also confirmed that all ATIs are encoded by the B and D genomes, with genes on the A genome appearing to be silenced (17, 40).

Although the individual ATI forms are encoded by single genes, the total ATI concentration shows polygenic inheritance due to the high number of isoforms.

Several studies have investigated the effects of the environment on inhibitor activities in different wheat genotypes, although most have measured total activity in unfractionated extracts rather than characterized ATI fractions components. Priya et al. (41) showed wide variation in the inhibitory activity of amylase and trypsin from two storage pests (the Coleopteran beetles *Tenebrio molitor* and *Rhyzopertha dominica*) and mammals (human salivary and porcine pancreatic amylases and bovine trypsin) in unfractionated extracts from 54 genotypes of bread wheat, with some lines having high activity against insect enzymes and low activity against mammalian enzymes. Similarly, Piasecka-Kwiatkowska et al. (42, 43) showed significant effects of genotype, harvest year, environment (precipitation), and interactions between these factors on inhibitory activity against amylases and trypsin from mammalian and insect sources (the wheat weevil *Sitophilus granaries*, the flour beetle *Tribolium confusum* and larvae of the flour moth *Ephestia kuehniella*) in genotypes of wheat, rye and triticale grown over 4 years. Significant effects of genotype and of genotype-by-year interactions, but not of year were also reported for bread wheat (44) using an assay based on trypsin inhibition whereas significant effects of genotype and location but not genotype-by-location interactions were reported for the amount of the CM3 subunit (which forms part of a tetrameric complex) determined by proteomic analysis of durum wheat (45).

## Analysis of ATIs

The selection and availability of appropriate reference materials are essential for the identification and quantification of ATIs which in turn are crucial to enable their monitoring in food systems and to provide well-characterized fractions to determine their bioactivity and adverse reactions in the human body. The presence of enzyme inhibitors other than ATIs should also be taken into consideration as they can contribute to measurements of inhibitory activity.

### Extraction From Flour and Food

Because ATIs are soluble in dilute salt solutions they can be extracted from milled grain or white flour using aqueous buffers as part of the widely used Osborne fractionation. However, they may also be present in other Osborne fractions, notably the prolamins (gluten proteins). Several procedures have been developed to prepare fractions highly enriched in ATIs. Lipids are first removed with butan-1-ol followed by petroleum ether (46) or with chloroform (47) at 4°C. A combined salt-soluble protein (albumin and globulin) fraction is then removed with salt-containing buffers such as 150 mmol/L NaCl (48), 50 mmol/L Tris-HCl, 100 mmol/L KCl, 5 mmol/L EDTA (49), or 50 mmol/L ammonium bicarbonate with the procedure repeated three times, resulting in a highly enriched ATI fraction (50). The extractability is also affected by the redox state: upon reduction CM proteins become insoluble in methanol (51).

Several studies have described the extraction of ATIs from processed foods, such as bread and raw and cooked pasta, using essentially the same methods as for flour. However, the extractability from processed foods can be affected by gelatinization of starch and denaturation of protein. For example, increasing the drying temperature of pasta reduced the extractability of ATIs (52, 53). This may limit quantification and hence comparison of the ATI contents of preparations used for determination of bioactivity.

Two main procedures have been developed for sub-fractionation of ATI extracts. ATIs can be precipitated sequentially by salting out with the addition of ammonium sulfate at concentrations between 0.4 and 1.8 mmol/L (48, 54). The second procedure exploits the solubility of inhibitors in chloroform/methanol (49) to separate them from metabolic proteins, although the fraction also contains some gluten proteins (51). A second extraction with saline removes gliadins and enriches ATIs, but other proteins of the prolamin superfamily (farinins and non-specific lipid transfer proteins) are still present (55). However, neither of these methods gives pure isoforms and additional purification steps are required such as chromatographic enrichment.

### Challenges for Analysis of ATIs by Mass Spectrometry (MS)

MS has the potential for very sensitive and selective detection of multiple ATIs. The full repertoire of ATIs can be identified by non-targeted discovery, using MS analysis after liquid chromatography and matching of MS/MS spectra to protein sequence databases. However, the development of targeted LC-MS detection is required to facilitate the routine use in crop and food science. For targeted detection, peptides are selected to represent the different types and isoforms of ATIs. Many variants and isoforms of ATIs have been reported in DNA and protein sequence databases with peptide sequences overlapping between multiple homeologues and isoforms (25). While some peptides are unique for one protein, others can occur in several related protein sequences. The choice of peptides for targeted LC-MS therefore determines the sensitivity and selectivity of the analysis.

The precision of MS analysis also depends on the availability of reliable genomic and proteome sequence databases from which incomplete and duplicated sequences are discarded and variant sequences are considered in the selection of the optimal target sequences. For example, the transcript TraesCS4B02G328100.1 encoding the CM3 protein (P17314) is associated with 146 variant alleles in EnsemblPlants database (56) whereas only a single genomic sequence is present in the Chinese wheat landrace Chinese Spring. Hence, the precise sequences of the isoforms in the species or genotypes being studied may not be present in the database and thus limiting their detection.

ATIs can also be quantified by determination of the molecular weight of full-length proteins by MALDI-TOF-MS (44, 57, 58).

### Establishment of Routine Analytical Methods

A combination of discovery and targeted proteomics by MS is clearly the most attractive approach for identifying and quantifying ATIs. Comparative results on the contents of monomeric, dimeric and tetrameric ATIs have been obtained by absolute and relative quantification (25, 59, 60). Especially, targeted MS analysis is characterized by very low limits of detection (LOD) with 0.1–3.9 μg peptide per g flour (60), while discovery driven proteomics has at least 10 times higher LOD (61). However, MS-based analysis may not be available to all researchers and alternative methods have been used to provide faster and cheaper analyses.

Routine biochemical analyses such as chromatographic or electrophoretic separation followed by UV detection give reproducible results, but the coverage is not complete (44). Another simple method is to determine the inhibitory activity of preparations toward target enzymes. However, the different specificities of the inhibitors (see section Types and Properties of ATIs in Bread Wheat) require the use of a range of substrate enzymes (41), and the assays may also detect inhibitors of other types (see Types and Properties of ATIs in Bread Wheat). These limitations, together with variation in experimental protocols, may account for the wide variation reported on inhibitory activities of ATIs in cereals (41, 44, 57). Consequently, the correlations between ATI concentrations and enzyme inhibitory activities are generally poor (57).

Immunological approaches are rarely used, mainly due to the lack of specific antibodies. Some studies have used serum antibodies from patients with wheat allergy, since ATIs have been identified as allergen in IgE-positive patients (Table 2). A cell culture system has also been used to measure ATI-mediated Toll Like Receptor-4 (TLR4) activation (discussed in section Effects of ATIs on Human Physiology and Pathology), as a marker for innate immunity related signaling induction by wheat extracts (50, 67).

**Table 2:** ATIs shown to elicit allergic responses.

**ATI**	**WHO/IUIS Allergen name**	**Allergen isoform UniProt accession number**	**Types of IgE-mediated allergy**	
			**Bakers' asthma**	**Food allergy**
0.28	Tri a 15.0101	D2TGC3	Yes^a^	No
	None	P01083	No	Yes^e^
0.19	Tri a 28.0101	Q4W0V7	Yes^a^	No
	None	P01085	No	Yes^b^, ^c^
	None	Q5UHI0	No	Yes^d^
	None	P01083	No	Yes^c^, ^e^
	None	Q5UHH6	No	Yes^c^
0.53	None	P01084	No	Yes^c^
CM1	Tri a 29.0101	C7C4X0	Yes^a^	No
	None	P16850	No	Yes^b^, ^c^
CM2	Tri a 29.0201	D2TGC2	Yes^a^	No
	None	P16851	No	Yes^b^, ^c^
CM3	Tri a 30.0101	P17314	Yes	Yes^b^, ^d^
	None	Q53YX8	No	Yes^e^
CM16	None	P16159	Yes	Yes^b^, ^c^, ^d^, ^e^
CMX1/CMX3	None	Q43723	No	Yes^c^
CM17	Tri a 40.0101	Q41540	Yes^f^	Yes^c^, ^d^

a *Sander et al. (62)*.

b *Pastorello et al. (63)*.

c *Sotkovský et al. (64)*.

d *Sotkovský et al. (65)*.

e *Tundo et al. (24)*.

f *Sander et al. (66)*.

To date, no detailed comparisons of ATI contents and either enzyme inhibition or TLR4 activation have been reported, and little is known about the role of factors such as glycosylation or oligomerization.

## ATIs in Other Types of Wheat and Related Cereals

**Figure 3:**
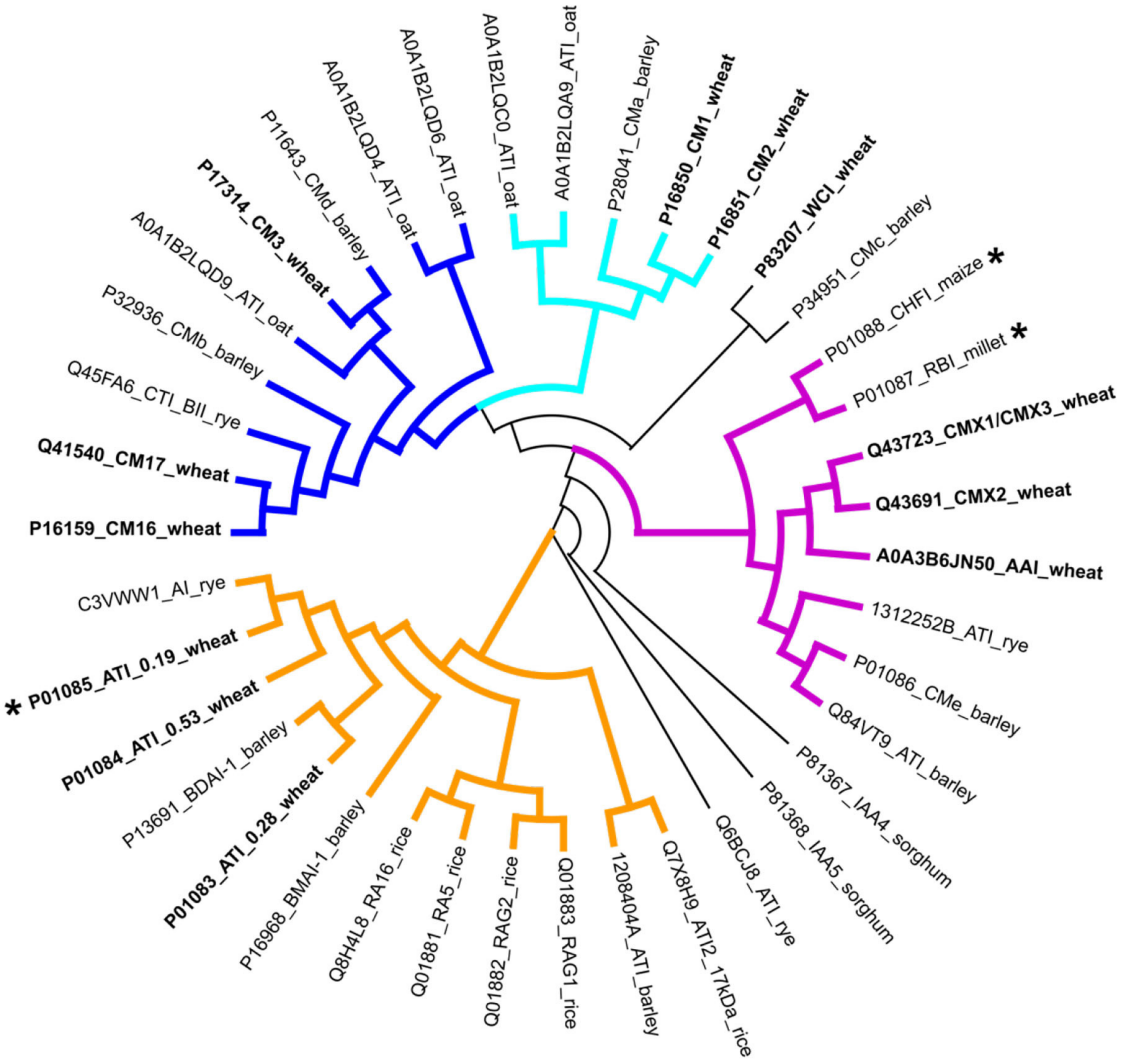
Phylogenetic analysis of ATIs in cereal species with wheat ATIs shown in bold. The sequence conservation of the ATIs is very low, but comparison of the three ATIs for which 3D structures have been determined (indicated by stars) shows that structural conservation is high. The coloring of the subgroups follows that used in Figure 1: blue and cyan, heterotetrameric; purple, trypsin/bifunctional inhibitors; orange, monomeric and homodimeric). Software used: MEGA X (12) (Muscle for alignment preparation and Maximum likelihood for phylogeny analysis), and Dendroscope (13).

Proteins of the ATI family can be identified in protein sequence databases by the presence of specific sequence motifs, termed the PROSITE signature (68) PS00426 or the PRINTS pattern PR00808. The ATI family (IPR006106) in the InterPro classification database (69) of protein families currently contains over a thousand protein accessions of which 35 are curated. These include accessions from wheat (19), barley (10), rice (7), sorghum (2), maize (1), and millet (1). It is clear therefore that ATIs occur widely in grass species including other wheat species, close relatives of wheat and other cultivated cereals (Figure 3).

Rogniaux et al. (59) used targeted MS analysis to determine peptides corresponding to five ATIs (0.19, 0.28, CM1, CM2, and CM3) in seven varieties from three wheat species (einkorn, durum wheat and bread wheat). 0.19 and CM1 were exclusively detected in bread wheat whereas 0.28, CM2, and CM3 were detected in all species, although at a much lower level in einkorn. Geisslitz et al. (60) quantified 13 ATIs including the predominant 0.19, 0.28, 0.53, CM1, CM2, CM3, CM16, and CM16 in eight genotypes of five wheat species (bread wheat, durum wheat, spelt, emmer and einkorn) which were cultivated at three locations in Germany. Similar contents were detected in the hexaploid and tetraploid wheats, while they were low or absent in diploid einkorn (which fits in with the observation of low contribution of ATIs from the A genome in hexaploid bread wheat; see sections Introduction and Genetic and Environmental Control of ATIs in Bread Wheat). Furthermore, the ATI content was much more influenced by genotype than by environment (see also section Genetic and Environmental Control of ATIs in Bread Wheat).

Consistent with the low ATI concentrations determined by MS (44, 60), einkorn showed no inhibition of Coleopteran insect or mammalian α-amylases (70–74) and the lowest TRL4-activating potential compared to other wheat species (50). However, einkorn inhibited α-amylase from Lepidopteran insects (72) and contains a trypsin inhibitor similar to rye and barley (70) which may explain the high trypsin inhibitory activity (44). Wild diploid wheats (*T. urartu* and *T. boeoticum*) showed contrasting results with either low or no α-amylase inhibition (70–74). Diploid *Aegilops* species with the D genome differed greatly from bread wheat in their α-amylase inhibitor pattern and their inhibitory activity (70, 73). Some tetraploid wheats, such as durum wheat (60), Persian wheat and Armenian wild emmer (71), lack monomeric 0.28. However, spelt, durum wheat and emmer all contain the potent TLR4 activators 0.19 and CM3 (45, 54, 58).

Hexaploid and tetraploid wheat differ in their proportions of monomeric, dimeric and tetrameric ATIs: the proportions of tetrameric, monomeric, and dimeric ATIs are similar for hexaploid wheat whereas the proportion of tetrameric CM-type inhibitors is higher in tetraploid wheats (60).

Barley grains contain ATIs which are similar to those in wheat, occurring in monomeric (BMAI), dimeric (BDAI) and tetrameric (BTAI-C) forms with masses of 10,000 to 16,000 (75) and sharing over 80% sequence identity with homologs in wheat (i.e., BTAI-CM and WTAI-CM, BDAI and 0.19/0.53) (76). All have inhibitory activity toward α-amylase from Coleopteran insects, but little or no activity against human salivary α-amylase (77, 78). Rye grain contains dimeric inhibitors highly homologous to the wheat dimeric 0.19 and 0.53 inhibitors (79, 80) and a trypsin inhibitor (81), but no tetrameric forms have been reported. Seventeen ATI-like proteins which exhibit up to 60% similarity with wheat CM proteins have been reported in oats (82). ATI-enriched extracts from barley and rye showed TLR4 activation comparable to wheat, which was not detected in extracts from oats and other non-gluten containing cereals (50). In this study hexaploid bread wheat induced higher ATI inflammatory bioactivity compared to ATI-enriched fractions from diploid (einkorn), tetraploid wheat (emmer, Khorasan wheat), and hexaploid spelt.

## Effects of ATIs on Human Physiology and Pathology

Data from *in vitro* and animal studies indicate that ATIs may play roles in the initiation of CD and non-intestinal auto-immune diseases. The proposed mechanisms are activation of the adaptive and innate immune systems, disruption of the intestinal barrier function and intestinal as well as extra-intestinal inflammation. In addition, ATIs have also been identified as potent triggers of IgE mediated allergic responses such as bakers' asthma. NCWS has recently been suggested to be a non-specific, non-IgE mediated type of a gut associated allergy in which ATIs may play a prominent role. However, only limited information is available about the immunogenic sequences of ATIs (83).

**Figure 4:**
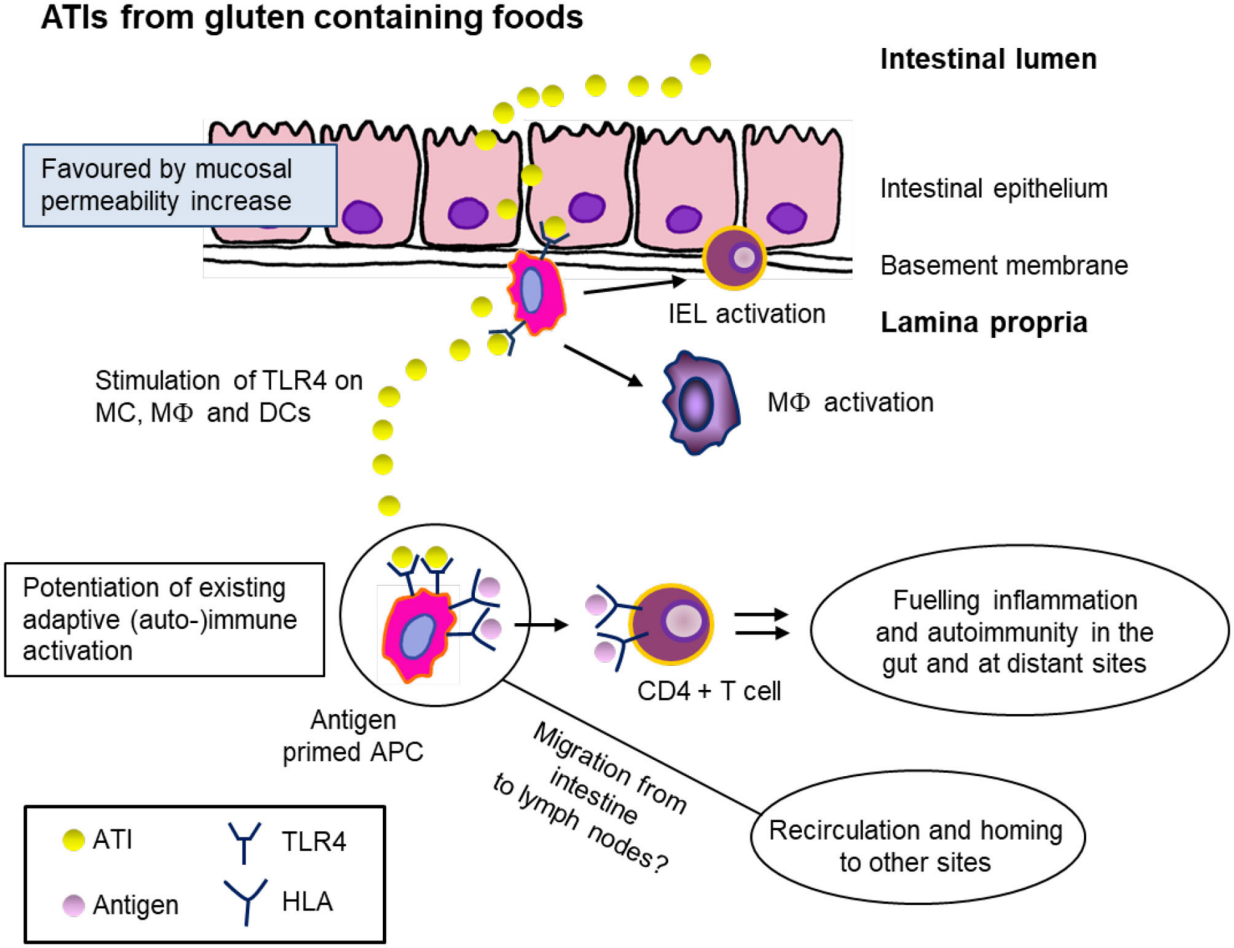
Hypothetical mechanism of ATI bioactivity as innate triggers of intestinal and extraintestinal immune activation after ingestion of gluten containing foods (e.g., wheat, spelt, rye, and barley). ATIs pass the intestinal epithelium as intact proteins and stimulate the toll-like receptor 4 (TLR4) on monocytes (MC), macrophages (MΦ), and dendritic cells (DC). This leads to potentiation of existing adaptive immune activation and an increase of antigen-presenting cells (APC). These serve as adjuvants for an ongoing adaptive T-cell response and intensify chronic and autoimmune diseases. It is supposed that the APC occur at extraintestinal sites, e.g., mesenteric lymph nodes. IEL, intraepithelial lymphocyte; HLA, human leukocyte antigen. Figure modified from Schuppan et al. (85).

As *in vivo* human pathological studies are lacking, caution is required when extrapolating results from *in vitro* and animal studies. In addition to the possible presence of impurities in ATI extracts, it is necessary to consider several other factors. (a) Wheat is normally consumed after processing which may include fermentation of dough (by yeast or sourdough culture), exposure to heat and high humidity (cooking, baking, extrusion) under various pH or ionic strength conditions. (b) Ingested food is exposed to gastric acid and bile acids [bile is known to affect proteolytic enzymes (84)] and proteolysis by pancreatic enzymes. (c) Lactobacilli present in the intestine may secrete proteolytic enzymes capable of degrading ATIs (6, 55). As a consequence, the resistance of extracted ATIs to proteolysis in *in vitro* digestibility studies may not reflect the situation *in vivo*. An overview of our current knowledge is presented below and a schematic representation of the proposed mechanisms given in Figure 4.

### *In vitro* and *in vivo* Animal Studies Addressing ATI Bioactivity

Junker et al. (67) showed that ATIs, in particular CM3 and 0.19, induce an innate immune response and activate monocytes, macrophages and dendritic cells *in vitro* via the TLR4-MD2-CD14 complex with a subsequent release of pro-inflammatory cytokines. The effect was not modified by *in vitro* pepsin-trypsin digestion. Furthermore, ATIs, as shown for CM3, can interact directly with the TLR4 receptor (86). Activation of TLR4 by release of IL-8 in monocyte/macrophage cell lines has been confirmed for ATI extracts derived from a wide range of foods, especially of gluten-containing cereal foods and being (in part) retained after food processing (50).

Administration of preparations enriched in ATI to non-obese diabetic/DQ8 mice over 2 weeks triggered intestinal intraepithelial lymphocytosis and barrier dysfunction in the absence of overt inflammation or mucosal damage (6). The presence of ATIs in the large intestine was shown to modify microbiota composition and metabolism. For example, decreases in *Lactobacillus* and the *Firmicutes/Bacteroidetes* ratio were observed after exposure to ATIs (6). In line with these observations, transplantation of microbiota from feces of mice fed ATI-enriched diets into mice fed on control diets increased the severity of colitis indicating a direct inflammatory stimulus of intestinal microbiota. Pickert et al. (87) suggested that ATI-associated dysbiosis and ATI-induced TLR4 activation are likely to occur simultaneously and may synergistically promote the overall inflammatory reaction and intestinal barrier function.

Although some studies have been carried out on inflammatory responses to wheat consumption, the test foods used or samples studied contained many other components in addition to ATIs and it is therefore not possible to conclude that the observed effects are exclusively caused by ATIs (88). This is supported by the observation that the major non-gluten proteins recognized by IgG and IgA antibodies from CD patients were ATIs, serpins, globulins and two groups of gluten-related proteins termed purinins and farinins (8).

Finally, it should be noted that the effects of ATIs on gut microbial diversity and metabolism can be bidirectional. On the one hand, as outlined above, the microbiota is influenced by the presence of ATIs. On the other hand, selected *Lactobacillus* strains from sourdough and human intestine were shown to express proteases able to degrade ATIs and thereby reduce inflammatory and immune responses (55, 89). In addition to potentiating intestinal inflammation, ATI extracts also increase liver and adipose tissue inflammation, liver fibrosis and insulin resistance in murine models of non-alcoholic steatohepatitis (90), and exacerbation of the pathological hallmarks of Alzheimer's disease in 5xFAD mice (91). These observations and the critical points listed above clearly indicate a need to study the effects of pure and well-characterized ATIs in relevant human cohorts suffering from inflammatory diseases.

### Coeliac Disease (CD)

Several authors have suggested that ATIs play a role in the etiology of CD by eliciting an innate immune response (67, 89). They suggest that ATIs may reduce the digestion of gliadins, leading to higher levels of digestion-resistant immunogenic gliadin peptides passing the small intestinal gut epithelium, and also potentiate the initiation of CD by enhancing the release of pro-inflammatory cytokines and chemokines which may act synergistically to gluten in causing CD.

### Non-coeliac Wheat Sensitivity (NCWS)

NCWS is a condition characterized by intestinal and extra-intestinal symptoms related to the ingestion of wheat and other “gluten-containing” cereals in patients in whom CD and wheat allergy had been excluded and who have symptomatic improvement on their withdrawal (92). Early studies led to the conclusion that gluten was responsible for the symptoms, but these studies used either wheat-based foods (93) or foods with added gluten (94), both of which contain other wheat proteins (as discussed above). In addition, wheat also contains FODMAPs (Fermentable Oligosaccharides, Disaccharides, Monosaccharides, and Polyols) which induce distress in sensitive individuals due to gas produced by fermentation. Consequently, other components consumed in wheat-containing foods may play a role, justifying the term NCWS for this condition. A follow-up study by Biesiekierski et al. (7) addressed this issue and observed no effects of added vital wheat gluten in patients with self-reported NCWS after dietary reduction of FODMAPs. Because this study used isolated gluten, which is known to contain ATIs (50), it can be inferred that neither ATIs nor gluten proteins were causative agents. Whereas, Vazquez Rogue et al. (93) concluded that gluten (accompanied by ATIs) alters bowel barrier functions in patients with diarrhea-predominant IBS (IBS-D), Biesiekierski et al. (7) observed no change in permeability. Other observations lead to the conclusion that the degree of increased small bowel permeability in NCWS patients is low compared with that of CD patients and healthy controls (95). A recent pilot study in irritable bowel syndrome (IBS) patients with self-reported NCWS demonstrated no significant differences in markers of low-grade inflammation and gastrointestinal symptoms when consuming bread containing reduced amounts of dimeric and tetrameric ATIs as a result of sourdough fermentation (96).

No controlled human interventions have been carried out with well-characterized purified compounds isolated from processed wheat-containing foods. Consequently, the potential effects of ATIs on the pathophysiology in NCWS remain unclear. Overlaps in symptoms with CD, wheat allergy and IBS are complicating factors and there are at present no established biomarkers to diagnose NCWS (92). Recent reviews have highlighted the clinical research challenges to help overcome in this impasse (85, 97, 98). Note that self-reported diagnosis plays a significant role in the perceived impact of NCWS.

### Wheat Allergy

Allergy to wheat occurs in two forms: (a) as inhalant or contact occupational allergies to flour (the former known as bakers' asthma) and (b) as classical food allergy triggered by ingestion of wheat proteins, including several ATIs (99, 100) (Table 2). The prevalence of wheat food allergy is relatively low (0.25%); it occurs mostly in children that overgrow this allergy. Note also that about 30 wheat proteins may cause IgE-related sensitization, however without any clinical relevance [reviewed in Gilissen et al. (101)]. According to Pastorello et al. (63) the allergenic potential of ATIs is not reduced by cooking for 5 min at 100°C. However, ATIs do not appear to trigger the rare, but most severe allergic response to wheat consumption, wheat-dependant exercise-induced anaphylaxis, which is triggered by gluten proteins.

By contrast, ATIs appear to be the most potent activators of allergic airway responses, such as bakers' asthma (62, 63) which is the second most common occupational trigger of asthma in the UK (https://www.hse.gov.uk/statistics/causdis/asthma.pdf), with glycosylated forms being particularly active (Table 2). It is therefore important to reduce flour dust in mills and bakeries.

The addition of ATIs to the diet was shown to potentiate allergic airway inflammation *in vivo* in OVA-challenged mice (102) and IgE-dependent allergic airway inflammation *in vivo* in humanized mice to which peripheral mononuclear blood cells from individuals allergic to grass and/or birch pollen and non-allergic individuals had been transferred (103). In this study, mice were placed on a diet free of gluten and ATIs for 3 weeks after which they were challenged by inclusion of a source of ATIs in the diet. Since no ATIs could be detected in blood, it was suggested that the potentiation of inflammation and increased levels of IgE after exposure to ATIs resulted from myeloid cells which were activated in the intestinal mucosa and migrated to the periphery/lungs. These observations require confirmation by studies in humans.

### Effects of ATIs on Food Digestion

Extracts from wheat flour (which contain ATIs and other inhibitors) have been shown to inhibit α-amylase activity and slow the release rate of glucose from starch during digestion resulting in modestly reduced glycemia and insulinemia (104–106). It is possible that ATI-induced inhibition of trypsin *in vivo* may result in increased levels of non-digested bioactive wheat proteins which may trigger immune responses and inflammation, but at present there is no evidence for this.

## Modifying ATI Content and Bioactivity in Grain and Food

Dietary exclusion of wheat-based foods to avoid pathogenic effects can result in depletion of essential dietary components such as fiber, proteins and minerals. Such exclusion can therefore be avoided by using plant breeding strategies to remove ATIs from grain or to apply processing strategies to inactivate them in foods.

### Manipulating the Content of ATIs in Grain

Two approaches can be used to reduce the amount or activity of ATIs in plants, but both require more detailed knowledge of the roles of individual proteins. The first is to exploit genetic variation in the content and composition of ATIs in wheat species (as discussed above). Variation between genotypes may also occur, as described for coeliac-toxic gluten proteins (107), but this has not been demonstrated for ATIs.

The second approach is to use mutagenesis or other technologies, including gene editing, to disrupt ATI genes. Transgenic lines of bread wheat silenced for CM3, CM16, and 0.28 ATI genes were produced using RNA-interference (108) while marker-free genome editing using CRISPR-Cas9 has been used to produce both small mutations and large deletions in CM3 and CM16 ATI genes in durum wheat (109). Mutations induced by gene editing are considered as GM in the EU, which effectively blocks their commercial exploitation, but they are not considered as GM in other countries (110).

Gene-edited plants can also be used to provide material to determine structure-function relationships of ATIs and to identify gene targets for mutation breeding. Mutation breeding has been used by plant breeders since the mid-20th century and is now facilitated for bread and durum wheats by the availability of TILLING (Targeting Induced Local Lesions IN Genomes) populations in which the exomes have been sequenced (111).

### Modification of ATIs by Food Processing

The effects of food processing on enzyme inhibition by ATIs are relatively easy to study, but the results are inconsistent. Whereas, enzyme inhibition has been reported to be increased in white bread (112) and to remain high in commercial wholemeal bread (113), it has also been reported to decrease during baking of bread (113–115) and boiling of pasta (113, 114). Inhibitory activity of the crust may also result from dusting of the loaves with flour prior to baking (113, 115). These conflicting results require further investigation.

Immunoassays generally require proteins to be soluble, which is a challenge for assaying ATIs in food because processing may result in protein denaturation, cross-linking, glycation and insolubility. No binding of IgE from serum of patients with allergy to wheat proteins including ATIs was observed after drying and cooking of pasta (116), or after the pasta was proteolytically digested *in vitro* (52, 116). However, ATIs were still detected by staining after electrophoresis of proteins from cooked pasta, indicating that processing resulted in a loss of epitope recognition (116). By contrast, one study showed that severe drying conditions induced molecular rearrangements of proteins leading to the formation of large protein aggregates which may have contributed to a moderate decrease in the *in vitro* protein hydrolysis and increase in the residual *in vitro* allergenicity (53).

ATIs have been reported to survive the *in vitro* digestion of raw pasta, but not of cooked pasta (117). Nevertheless, when 20 patients with IBS were challenged with cooked pasta, almost all had symptoms, and half had IgE against a range of wheat proteins with ATIs being the most prominent allergenic proteins (118). Several other *in vitro* studies have shown reduced immunogenic activity in processed foods including various bread types, toasted bread, pasta, and biscuits (50, 118–120). However, the reduced allergenic reactions observed *in vitro* could result from steric hindrance of epitopes in the denatured proteins rather than destruction, allowing them to be exposed after digestion.

Targeted enzymatic treatment of ATIs, fermentation and other approaches may be used to mitigate the effects of ATIs. Sourdough fermentation is known to have proteolytic activity resulting in digestion of ATIs (121), especially tetrameric ATIs, resulting in lower inflammatory activity (122).

Salt-soluble proteins including ATIs are also hydrolysed during fermentation by typical sourdough lactobacilli (*L. plantarum, L. brevis, and L. sanfranciscensis*) (121, 123). Understanding the mechanisms of action of the bacteria could therefore mitigate ATI pathology by processing. It should also be noted that although ATIs are water-soluble, they are also present in commercial gluten fractions that are widely used in food processing as technological aid (50).

## ATIs and Human Health: Where Do We Stand in 2021?

Wheat is an important staple food for humankind and has been consumed for millennia. Its nutritional quality and other impacts on health are therefore crucial for food security. Although most people can eat wheat-based products safely, ATIs from wheat are of concern for some groups of people.

### What Do We Know?

- Enzyme inhibitors are present in all forms of cultivated wheat, and in related wild species and cereals
- ATIs play a clear role in bakers' eczema and asthma and in food allergy.
- *In vitro* studies and *in vivo* studies in animals show that ATIs play a role in the pathogenesis of gut-related disorders.
- Food processing and plant biotechnology offers opportunities to reduce the amount and activity of ATIs in food.

### What We Do Not Know?

- The impact of ATIs and pure and well-defined isoforms on pathologies in humans are not clear, including mechanisms at the molecular level. *In vivo* studies on the pathogenesis of gut-related disorders by ATIs in humans are lacking.
- The relationship, if any, between ATI activity in humans and their enzyme inhibitory properties has not been established.
- The extent to which activity in humans varies between types and isoforms of ATI present in wheat and other species is not known.
- The impacts of food processing on the effects of ATIs upon consumption are not understood.

### The Challenges We are Faced With

- Developing accurate methods to quantify ATI isoforms: these are currently limited by the complexity of the mixture and the properties of the components, especially in processed foods.
- Providing sufficient quantities of pure ATI fractions and forms to determine activity in humans.
- Understanding the mechanisms by which ATIs impact human adverse reactions to wheat and related pathology, including the induction of the autoimmune process with consequent destruction of specific tissues.
- Understanding why the majority of consumers have no pathological responses.
- Developing methods to reduce active ATIs in foods without effects on food quality acceptability.

These challenges must be addressed, if wheat is to be accepted as a safe and nutritious food in the future.

## Author Contributions

SG, PS, FB, and PW: wrote the first draft of the manuscript and contributed to revising and editing the manuscript. SG, PS, MM, and CM: conceptualization and preparing of figures and tables. All authors writing parts belonging to their expertise, read, and approve the final manuscript.

## Conflict of Interest

The authors declare that the research was conducted in the absence of any commercial or financial relationships that could be construed as a potential conflict of interest.
